# The oviductal transcriptome is influenced by a local ovarian effect in the sow

**DOI:** 10.1186/s13048-016-0252-9

**Published:** 2016-07-22

**Authors:** Rebeca López-Úbeda, Marta Muñoz, Luis Vieira, Ronald H. F. Hunter, Pilar Coy, Sebastian Canovas

**Affiliations:** Department of Physiology, Veterinary Faculty, University of Murcia, Campus de Espinardo, 30100 Murcia, Spain; International Excellence Campus for Higher Education and Research (Campus Mare Nostrum), Murcia, Spain; Centro de Biotecnología Animal - SERIDA, Deva, Gijón, Asturias Spain; Sidney Sussex College, Cambridge, UK; IMIB-Arrixaca (Institute for Biomedical Research of Murcia), Murcia, Spain

**Keywords:** Pig, Oviductal transcriptome, Microarray, Ovariectomy

## Abstract

**Background:**

Oviducts participate in fertilization and early embryo development, and they are influenced by systemic and local circulation. Local functional interplay between ovary, oviduct and uterus is important, as deduced from the previously observed differences in hormone concentrations, presence of sperm, or patterns of motility in the oviduct after unilateral ovariectomy (UO). However, the consequences of unilateral ovariectomy on the oviductal transcriptome remain unexplored. In this study, we have investigated the consequences of UO in a higher animal model as the pig.

**Methods:**

The influence of UO was analyzed on the number of ovulations on the contra ovary, which was increased, and on the ipsilateral oviductal transcriptome. Microarray analysis was performed and the results were validated by PCR. Differentially expressed genes (DEGs) with a fold change ≥ 2 and a false discovery rate of 10 % were analyzed by Ingenuity Pathway Analysis (IPA) to identify the main biofunctions affected by UO.

**Results:**

Data revealed two principal effects in the ipsilateral oviduct after UO: i) down-regulation of genes involved in the survival of sperm in the oviduct and early embryonic development, and ii) up-regulation of genes involved in others functions as protection against external agents and tumors.

**Conclusions:**

Results showed that unilateral ovariectomy results in an increased number of ovulation points on the contra ovary and changes in the transcriptome of the ipsilateral oviduct with consequences on key biological process that could affect fertility output.

**Electronic supplementary material:**

The online version of this article (doi:10.1186/s13048-016-0252-9) contains supplementary material, which is available to authorized users.

## Background

Among all structures involved in the fertilization process, it is in the oviduct where the most decisive episodes for fertilization take place [1, 2]. Recently, the oviduct transcriptome has aroused the interest of different groups, including ourselves; these have studied changes in human [3, 4] and animal oviductal transcriptome profiles under different conditions [5–8], trying to elucidate the consequences for fertilization and early embryo development.

In contrast to extensive studies focused on interactions between ovary and endometrium, interactions with the oviduct appear to have been overlooked. A better understanding of the local regulatory interactions between ovary and oviduct could help us to make progress in livestock reproduction and also improve in vitro culture conditions used in assisted reproductive technologies (ART) [9].

Oviducts are influenced by the systemic circulation, that changes throughout the reproductive cycle [1], primarily due to the action of ovarian steroid hormones [10, 11] and prostaglandins [12–14]. In addition, local functional interplay between ovary, oviduct and uterus are also important [15, 16]. The existence of a local regulatory mechanism has been documented in women and animal species from anatomical studies and functional evaluation of blood distribution (Additional file 1: Table S1), and it is already known that small changes in concentrations of progesterone can affect the oviduct secretions such as amino acids and ions [17, 18], so it could be expected that unilateral ovariectomy (UO) would have consequences in the oviduct, but this question remains unexplored until now.

The objective of this study was to investigate differences in oviductal transcriptome caused by the presence or absence of the local effect of the ovary following UO, in a specific area of the oviduct (ampullary-isthmic junction) where fertilization and zygote formation occurs [19].

We employed an in vivo model in which no hormonal treatment (animals were in natural oestrus) or previous surgery were performed. The principal tool used to achieve our objective was the Porcine Gene Expression Microarray (ID 026440 Agilent Technologies, Madrid, Spain).

## Results

### Compensatory ovulation mechanism

The number of points of ovulation and ovarian weights at the first and second surgery were compared (Table 1). Unilateral ovariectomy had a significant effect on the number of ovulation points, which were 3 times higher in the ovaries obtained in the second surgery (7.6 ± 1.6 vs. 22.00 ± 2.4; *p* < 0.017). In contrast, removal of one ovary had no significant effect on the weight of the remaining ovary except for sow number 5 where ovary weight increased after ovariectomy. No other morphological changes were noted in the ovary or connective tissue.

**Table 1:** Animal data sheet

		First surgery		Second surgery	
Sow	No. of previous farrowings	No. Ovulated^a^ follicles	Ovary weight (g)	No. Ovulated^a^ follicles	Ovary weight (g)
1	3	8	11.97	25	10.98
2	2	10	7.79	23	5.06
3	4	2	8.90	25	11.02
4	3	11	13.89	Not ovulated	11.72
5	2	7	5.88	15	48.07

^a^Paired-samples *t*Testanalysis for mean ± s.e.m. comparisons showed significant differences (*p* < 0.01) for the No. of ovulated follicles betweenthefirst (7.60 ± 1.6) and secondsurgery (22.00 ± 2.4)

### Genes differentially expressed between ovariectomized and control oviducts

Bioinformatics analysis of the microarray data showed 18 DEGs (with a fold change > 2 and a false discovery rate of 10 %) in ipsilateral oviduct (respect to ovariectomy) vs. contraleral oviduct. Twelve genes were up-regulated (*MSC*, *IGHA1*, *MIS18BP1*, *SEMA3B*, *AKAP5*, *LZTS3*, *MTOR*, *TMEM184B*, *ALOX12*, *RPP40*, *PRAP1* and *PTH1R*; Table 2). In contrast, six genes were down-regulated (*RPL37A*, *SEPP1*, *ITIH2*, *CFH*, *RBBP6*, *SAL1*; Table 2). Quantitative RT-PCR results for 3 selected genes (*ALOX12*, *CFH* and *SAL1*) confirmed microarray results (up- or down-regulation; Table 3), but differences in the fold change were detected, as typically have been described in other reports.

**Table 2:** Genes up- or down-regulated by ovariectomy

Gene bank accession	Symbol	Entrez gene name	Fold change
AK350490	*MSC*	musculin	2.88
AB194101	*IGHA1*	immunoglobulin heavy constant alpha 1	2.75
XM_003353476	*MIS18BP1*	MIS18 binding protein 1	2.61
XM_001927855	*SEMA3B*	semaphorin 3B	2.58
XM_001924844	*AKAP5*	A-kinase anchor protein 5-like	2.55
AK392511	*LZTS3*	leucine zipper, putative tumor suppressor family member 3	2.27
XP_003127632	*MTOR*	mechanistic target of rapamycin (serine/threonine kinase)	2.19
XM_001924228	*TMEM184B*	transmembrane protein 184B	2.17
NM_213931	*ALOX12*	arachidonate 12-lipoxygenase	2.14
AK233279	*RPP40*	ribonuclease P/MRP 40 kDa subunit	2.13
CN156206	*PRAP1*	proline-rich acidic protein 1-like	2.08
NM_214382	*PTH1R*	parathyroid hormone 1 receptor	2.02
XM_003133119	*RPL37A*	60S ribosomal protein L37a	−3.46
NM_001134823	*SEPP1*	selenoprotein P, plasma, 1	−3.43
NM_213903	*ITIH2*	inter-alpha-trypsin inhibitor heavy chain 2	−3.28
NM_214281	*CFH*	complement factor H	−3.05
XM_003124540	*RBBP6*	retinoblastoma binding protein 6	−2.65
NM_213814	*SAL1*	salivary lipocalin	−2.05

**Table 3:** Validation of microarray results using real-time RT-PCR

	Microarray		qPCR GAPDH		qPCR ACTB	
Genes	Mean fold changes	*P* value	Mean fold changes	*P* value	Mean fold changes	*P* value
*ALOX12*	2.14	0.042	11.58	0.049	6.55	0.047
*CFH*	−3.04	0.005	−8.11	0.028	−5.43	0.042
*SAL1*	−2.05	0.02	−9.04	0.032	−5.5	0.048

### Pathways, networks and biofunctions from Ingenuity Analysis

**Fig. 1:**
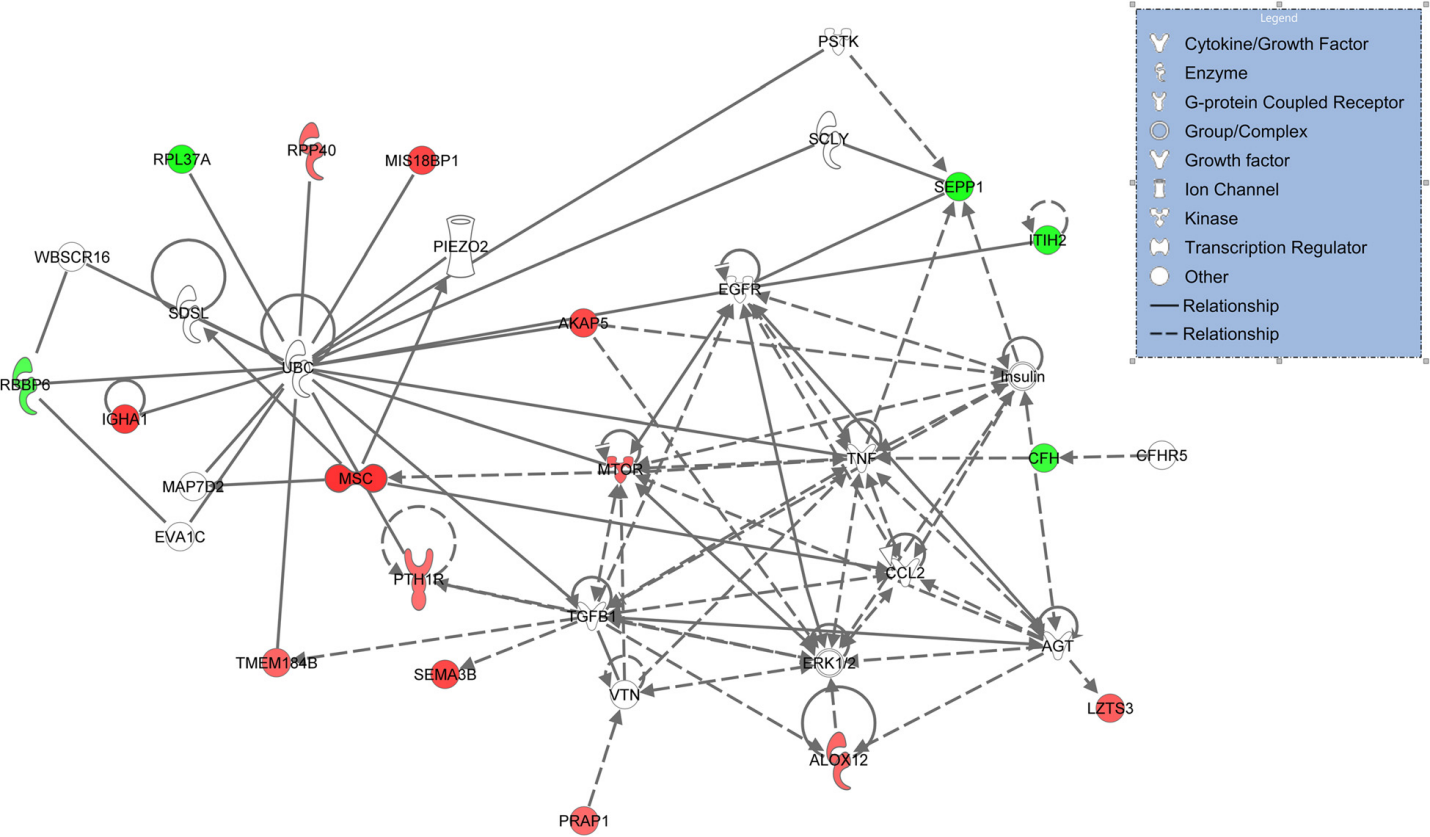
Interactome of functional associations among genes (up-regulated in red; down-regulated in green) included in the Network by Ingenuity Pathway Analysis

IPA software generated a single functional network able to integrate 17 of the 18 selected genes (12 up and 5 down-regulated genes; marked red and green, respectively) as shown in Fig. 1, with a very high score (51 score value). The gene *SAL1* is the only gene not included in the network because the Ingenuity database does not recognize it. Associated top network functions were: cellular growth and proliferation, cellular development, cell-to-cell signalling interaction, tissue development and lipid metabolism. Interestingly, *UBC*, *TGFB1*, *ERK1/2*, *TNF* and *INSULIN* are candidates to be involved in the cited functions as inferred from the gene network. Although these genes were not found to be differentially expressed in the microarray analysis, they are targeted by several DEGs.

The molecular and cellular functions related to DEGs (Additional file 3: Table S3) were cell death and survival (*n* = 9), cell morphology (*n* = 7), small molecule biochemistry (*n* = 7), amino acid metabolism (*n* = 4) and carbohydrate metabolism (*n* = 4). Acute Phase Response Signaling, cAMP-mediated signaling, Complement System, Hematopoiesis from Pluripotent Stem Cells and CNTF Signaling were the most significant canonical pathways affected (Additional file 3: Table S3).

### Predicted upstream regulators

**Fig. 2:**
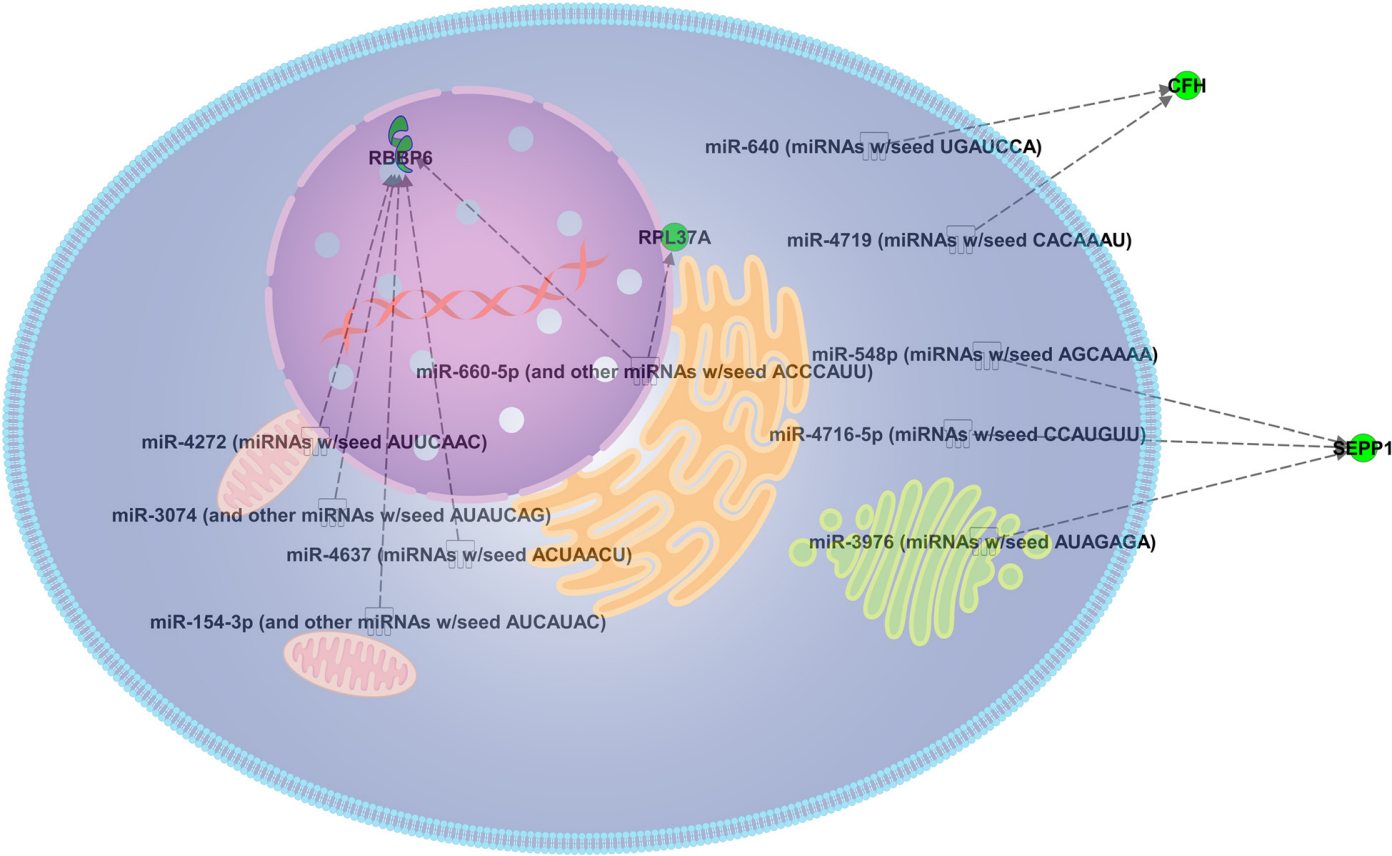
Predicted miRNAs that could act as upstream regulators of down-regulated genes in unilaterally ovariectomized sows

**Fig. 3:**
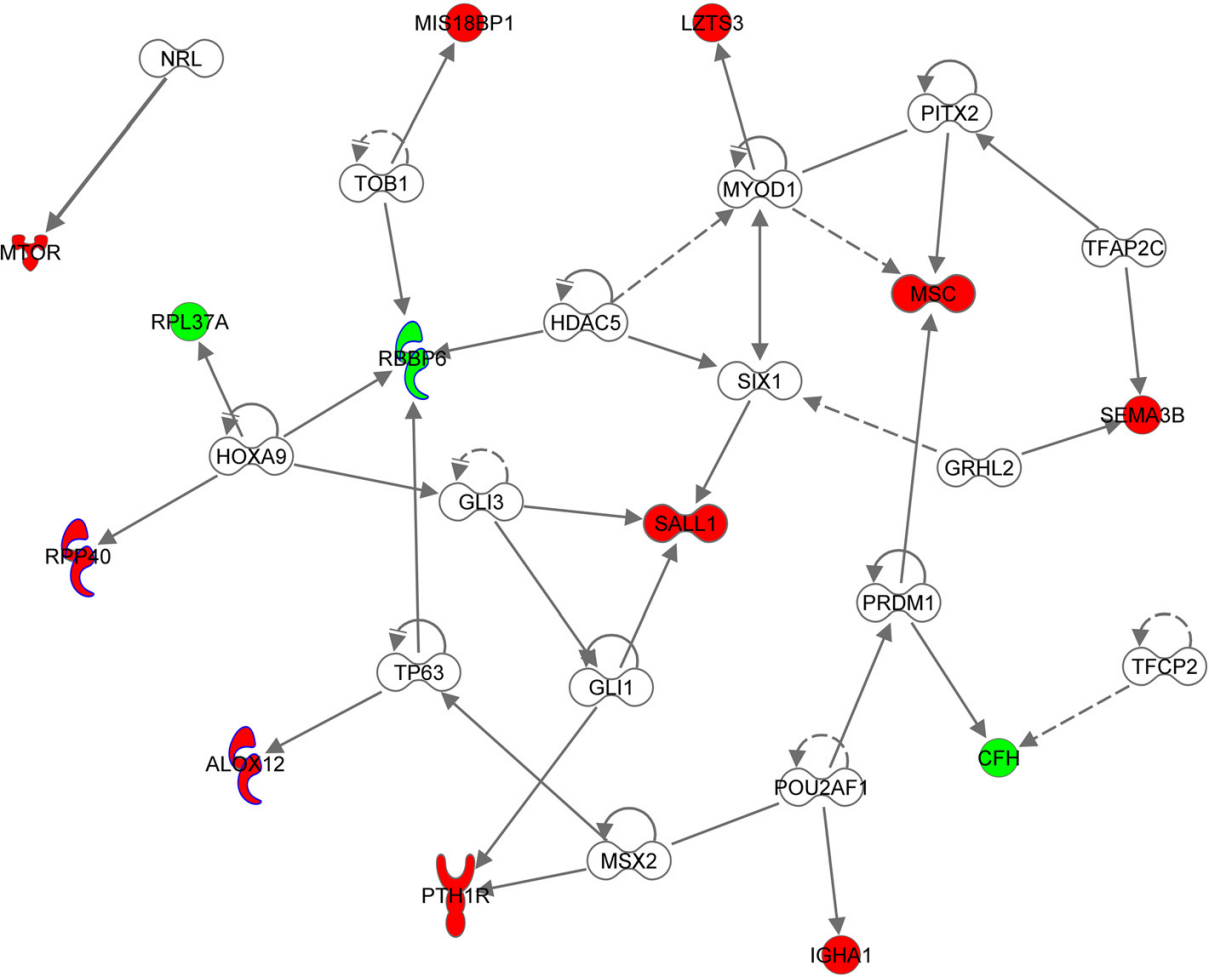
Network of upstream regulators factors of differential expressed genes (up-regulated in red; down-regulated in green) in unilaterally ovariectomized sows

We interrogated IPA for upstream microRNA (miRNA) regulators of down-regulated genes in this study. Results showed 9 predicted miRNAs that could act on *CFH*, *SEPP1*, *RPL37A* or *RBBP6*. Additionally, one miRNAs (miR-680-5p) was identified as regulator of *RBBP6* and *RPL37A* (Fig. 2). Moreover, we also obtained a prediction of other (not miRNAs) upstream regulators that could control genes that were identified as down and up-regulated genes in UO gilts. Results showed 15 factors that could act as upstream regulators (Fig. 3). Some factors could act simultaneously as a regulator of several genes, for example *HOXA9*and *TP63* are transcription factors which actually activate transcription. They were found as potential upstream regulators for the *RBBP6*, *RPP40*, and *RPL37A*or *ALOX12* and *RBBP6,* respectively. In addition, indirect interactions were frequently observed between upstream regulators. Ten from twelve genes were included in the same network of upstream regulator genes.

## Discussion

Classical studies had analyzed the consequences of UO and showed fewer litters and shorter breeding potential in specimens with single ovary (reviewed by [20]. Also, a shorter reproductive life span has been proposed as a consequence of compensatory follicular recruitment in women with UO [21, 22]. Our data confirmed an increase in the number of ovulation points (compensatory ovulation) in contralateral ovary after UO, as previously reported in different animal species [23–27]. Nonetheless, ovarian hypertrophy (anatomic compensatory effect) was only observed in one animal (sow 5; Table 1), probably because it does not occur immediately after the ovariectomy, similarly to other animal species (hamsters [28], guinea pigs [29] and rats [30].

Recently, transcriptomic profiling has been employed to understand and improve the knowledge about reproductive physiology in humans and animals. Human oviduct was analyzed and the transcriptome displayed the importance of factors related with immunomodulation, anti-angiogenesis or ion transport and secretions during transport of the embryos through the oviduct, which influence acquisition of implantation competence [3]. Information about the oviductal transcriptome in non-ovariectomized sows has been recently published by our own group [8]. Data from this report showed that insemination affects the expression of 26 genes (two-fold changes or more) involving in pathways related to inflammatory response, immune system, protein trafficking developmental disorder and cell-to-cell signaling. Also, subtle changes as for example the presence of X- or Y-sperm, or embryos influences oviduct functionality [5, 6]. However, the effect of UO in oviductal transcriptome has not been previously reported but we expected that could produce a moderate but key impact. The variability by obtaining paired samples from different animals (ovariectomized and non-ovariectomized) could mask this effect, especially when working with sows that are not inbreed animals such as mice, However, the experimental design that we used minimize the problem of animal-to-animal variability between the paired samples by using ipsilateral and contralateral oviducts from the same animal. Nonetheless, the use of control samples from animal without ovariectomy would allow to analyze the oviduct under physiological conditions, without any confounders, but it was not the aim of this study.

In our study, the specific objective was to investigate the local effect of the ovary in the oviductal transcriptome after UO. As we assumed that contralateral oviduct could be also influenced by UO (systemic effect) similar to the ovary that show ovarian compesantion, to analyze the isolated local effect of the ovary on the oviduct we used the contralateral oviduct as control sample, which have been under the same systemic effect but not under the ovarian local effect.

The differentially expressed transcripts in each pair of oviducts, ipsilateral and contralateral to ovariectomy, were analyzed by IPA to identify pathways influenced by UO. Although the number of genes that significantly changed their expression profile after UO was limited to eighteen (Table 2), a detailed analysis showed that UO affects oviduct functionality with up- or down-regulation of some factors involved in key process for reproduction. After UO, the oviduct adapts, altering its transcriptome, to a new situation in which it does not expect the arrival of oocytes due to the absence of the ovary.

Detailed analysis of the results revealed two main effects in ipsilateral oviductal transcriptome, maybe focused on saving energy and resources: i) down-regulation of genes involved in the survival of sperm in the oviduct and early embryonic development (*SEPP1*, *CHF* and *RBBP6*), and ii) oviduct focuses its resources to other ways such as the protection against external agents and tumors, up-regulating genes to immune response (*IGHA1*, *ALOX12* and *ITIH2*) and tumours suppressors (*LZTS3* and *SEMAB3*), which could be useful considering that fertilization is not expected in the ipsilateral oviduct after UO.

On the one hand, the expression of genes intended to facilitate the survival of sperm in the oviduct and early embryonic development were reduced. Among them, there is *SEPP1* which functions as an antioxidant in the extracellular space through selenium metabolism. In the oviduct, there is a fine tuned antioxidant system, regulated especially by glutathione peroxidases [31], and *SEPP1* could also participate in it. As occurs in the case of other selenoproteins (GPx-4), its expression in the oviduct is up-regulated by estrogens [32] and promotes activation of its antioxidant function [33–35] that could act to promoted sperm survival [36]. Results confirmed that *SEEP1* transcript level was directly affected by ovariectomy, probably through estrogen reduction that decreases *SEEP1* mRNA after UO.

Another down-regulated gene is *CFH*, a key cofactor of Complement 3, that in porcine protects sperm against complement attack in genital tracts [37], and its derivatives (C3b and iC3b) act as embryotrophic factors in early embryo development [38, 39], but whose functions are not necessary if fertilization will not happen in that oviduct by the absence of ovulated oocytes after UO. Similarly, *RBBP6*, whose function is proliferation and early embryo development [40, 41], was also down-regulated. Removing the ovary, ovulated oocytes will not be available to give rise to embryos so, this function is no longer necessary and both genes are down-regulated as our results would confirm.

Previous studies have shown that after UO there is a reduction in the number of secretory granules from the secretory cells of either tubal segments [42] since it is not necessary to prepare for gametes and embryo arrival. Therefore would be expected a down-regulation of genes related to oviductal secretion and regulated by estrogens, *SAL1* and *SEPP1* down-regulation was highlighted in this study. In sows, uterine *SAL1* is liable to be affected locally by embryo-produced estrogen/cytokines [43, 44]. Similarly, ovarian hormones–through local circulation- could also affect *SAL1* expression in the oviduct and would explain the observed down-regulation of *SAL1* in ipsilateral UO. The other estrogen modulated factor is *SEPP1* [45], which is involved in maternal to fetal transfer of selenium via the visceral yolk sac and the placenta [46], and it is not anymore necessary if the oviduct is not going to received zygotes.

On the other hand, after UO, oviduct focuses its resources to other functions such as the protection against external agents, up-regulating genes to immune response as *IGHA1*, *ALOX12* and *ITIH2*, and genes linked to tumour suppression such as *LZTS3* and *SEMAB3. IGHA1* is a steroid-regulate gene, that participates in antibacterial humoral responses through body fluids, and it may protect the oviduct from external contaminants similar to its function in the cervix [47]. *ALOX12* is involved in inflammation and immunity, and is related with menopause, a situation that somehow is partially simulated by UO [48–50]. Finally, *ITIH2* together with *MTOR* are two factors involved in the Acute Phase Response Signalling [51, 41]. This pathway, that also involved the participation of *IL-6* and *STAT3*, is related with inflammation.

*LZTS3* is a putative tumour suppressor family member, expressed in human oviduct [52], highly similar to *LZTS1* that inhibits cancer cell growth through the regulation of mitosis [53] and it is considered as a roadblock to reprogramming [54]. Bearing in mind that early embryos should undergo an extensive reprogramming and active cell growth, up-regulation of *LZTS3/LZTS1* could be considered incompatible with normal embryo development. However, in the context of UO, up-regulation of *LZTS3* could be understood as activation of a safeguard for tumour suppression in the oviduct. Another gene differentially expressed (up), and also involved in tumour suppression was semaphorin 3B (*SEMA3B*) [55]. Semaphorins have a role in angiogenesis, organogenesis, immune cell regulation and carcinogenesis [56–58].

Other genes differentially expressed in our study that could influence oviduct physiology are *PTH1R* and *AKAP5*, which are related to oviductal and uterine contractions [59, 60]. Although to the best of our knowledge *AKAP5* activity has not been described in the oviduct, we speculate that it could act similarly to the uterus. The up-regulation of these two factors involved in contractile activity would reveal increase motility in the ipsilateral ovariectomized oviduct. These contractions may targeted towards prevent the entry of sperm in case a mating or insemination should occur, favouring the entry of such sperm in the other oviduct in which the presence of oocytes is expected, increasing the possibilities of fertilization. This idea is supported by the fact that in a previous study by our group, *PTH1R* gene expression is reduced significantly with the arrival of sperm to the oviduct [8].

## Conclusions

This study demonstrates for the first time how an ovary can locally influence the oviductal transcriptome in the sow. In spite of a reduced number of genes changed, molecular and cellular functions related to these genes are important enough to influence oviduct functionality and, in the long term fertility. Overall, this study indicates the fine-tune regulation of gene expression in the oviduct focused on saving energy and resources, and emphasizes the important role that this small segment of the female tract plays in fertility, and also in pathologies as cancer.

## Methods

### Experimental design

Five sows were operated on to remove one ovary (first surgery) during the early luteal phase of the estrous cycle. A second intervention was made in which the remaining ovary and both oviducts were removed. The oviducts with ovary (*n* = 5; control) and oviducts of the contralateral side, without ovary, (*n* = 5; ovariectomized) were used for Gene Expression Microarray analysis. The experimental design chosen is the only possible way to assess the consequences that UO has on the own individual undergoing UO. In addition, this experimental design eliminates/minimizes the problem of animal-to-animal variability between the paired samples, which when working with large animal as sows, could be important because they are not inbreed animals such as mice.

### Animal care

The study was performed using multiparous sows without hormonal treatment or previous surgery. All the samples were collected from non-pregnant sows. Five sows (Landrace × Large White) under standard conditions of housing and feeding (water was provided ad libitum) were used. The care and manipulation of the animals were carried out in strict accordance with the ethical guidelines of Guiding Principles for the Care and Use of Animals (DHEW Publication, NIH, 80-23) and EC Directive 86/609/EEC for animal experiments. Surgery was performed under analgesic and anaesthetic protocols, and all efforts were made to minimize suffering.

### Estrous detection, ovariectomy surgery and tissue collection

All animals were monitored from weaning until they displayed signs of standing estrous. At this time, follicular size was evaluated by ultrasonographic scanning, confirming the existence of pre-ovulatory follicles. With all of animals in estrous, the first surgery was scheduled a week later to ensure that all sows had ovulated.

**Fig. 4:**
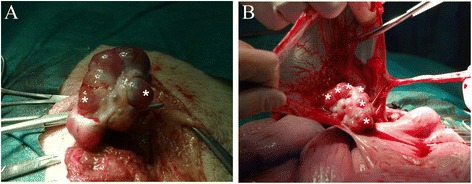
Details of the ovaries during the surgeries. **a** First surgery (unilateral ovariectomy), shows the removal of the ovary of sow with the minimum possible invasion. **b** Second surgery, shows removal of the oviduct on the side not ovariectomized. Compensatory ovulation can be observed. Asterisks show the points of ovulation

During the first surgery one ovary was removed (unilateral ovariectomy), with the minimal trauma (Fig. 4a). After surgery, the animals were monitored until next estrous detection. The second surgery proceeded after the next estrous, when the contralateral ovary and both oviducts were removed within 20 min after sedation (Fig. 4b). Preoperative anesthetic and sedation of animals was carried out following the protocol described previously by García-Vázquez et al. [61].

The ovaries were weighed and the number of recently-ovulated follicles recorded. Samples of oviductal tissue from the ampullary-isthmic junction (sample size > 1 cm including the end of the ampulla) were taken and immediately frozen in liquid nitrogen for later mRNA extraction, cDNA reverse transcription and further study by microarray (no flushing was used to avoid transcriptome alteration).

### RNA extraction and quality assessment

Total RNA was extracted from ten oviducts using the ‘TRIzol method’ (Life Technologies Inc., Gaithersburg, MD) following the manufacturer’s protocol. RNA samples were verified qualitatively and quantitatively by RNA 6000 Nano LabChip® kit and the Agilent 2100 Bionalyzer (Agilent Technologies, Santa Clara, 150 CA) respectively. Total RNA from ten oviducts of sows was obtained, two oviducts for each animal, control (*n* = 5) and ovariectomized (*n* = 5). All samples showed a RIN number above 7.0 and were used for microarray analysis.

### Microarray analysis procedures

The microarray experiments (sample preparation, hybridization and scanning) were performed using Agilent Technologies (Madrid, Spain) in accordance with the manufacturer’s instructions. For each sample, 300 ng of total RNA were first converted to cDNA using T7-oligo (dT) primers, then generating amplified cRNA using T7 RNA polymerase and labelled with Cy3 (Quick-AMP labelling kit, Agilent Technologies, Madrid, Spain). The labelled cRNA was purified to remove unincorporated Cy3 (Qiagen). To avoid interferences during hybridization, cRNA was fragmented to an optimal size of 35 to 200 bases long. This process was monitored by Agilent 2100 Bioanalyzer technology. All the samples were hybridized in Porcine Gene Expression Microarray (ID 026440 Agilent Technologies, Madrid, Spain) for 17 h of incubation at 65.8 °C with constant rotation, followed by a two-step microarray wash of 1 min in two buffers. Subsequently, hybridized chips were scanned by the Axon 4100A scanner (Molecular Devices, Sunnyvale, CA).

Microarray image analysis and RAW data were generated using GenePix Pro6.0 software. Statistical analysis was performed using BioConductor for R with the packages Linear Models for Microarray data (LIMMA), Marray, pcaMethods, EMA and RankProd. After background subtraction with the method “Normexp” from the LIMMA package, with an offset of 10, the data were normalized using “quantile” normalization. The screening criteria for the differences in gene expression between control and ovariectomized oviducts were established in a ≥ 2 fold change and a false discovery rate of 10 %. The basic local alignment search tool at the National Centre for Biotechnology Information (www.ncbi.nlm.nih.gov/blast) was used to identify the different genes. The mean fold change was calculated for those genes identified in more than one probe set.

### Ingenuity pathway analysis

Ingenuity Pathway Analysis (IPA, Ingenuity Systems, Redwood City, CA) was used to classify the selected differentially expressed genes into functional pathways, and were further annotated and classified based on the Gene Ontology (GO). List of differentially expressed genes with associated fold change values was uploaded into IPA for functional analysis. The data were used to generate an interaction network (Fig. 1). In the network, red indicates gene up-regulated and green depicts down-regulated genes. Symbols for each gene are presented according to molecular functions and type of interactions. In addition, predicted upstream regulators of gene differentially expressed in our study were identified using IPA tools.

### Microarray validation by Real-time Reverse Transcription-PCR (RT-PCR)

To validate microarray data, real-time RT-PCR was performed for three genes (*ALOX12*, *CFH* and *SAL1), a* number of genes similar to previously used in other reports. Results were normalized using two genes *ACTB* and *GADPH.* Forward and reverse primers were specific for each gene (Additional file 2: Table S2) and were designed with its sequence interrupted by intronic regions to avoid possible contamination by genomic DNA. First, 1 μg of each sample of total RNA (control *n* = 5, ovariectomized *n* = 5) was reverse transcribed using the MultiScribe Reverse 200 Transcriptase (Life Technologies, Inc.). The qPCR was performed in a thermal cycler StepOne™ (Applied Biosystems, Foster City, CA) using 5x HOT FIRE Pol EvaGreen qPCR Mix Plus (Solis BioDyne, Tartu, Estonia) containing EvaGreen, an alternative to SYBR Green but with similar properties, and ROX for normalize. Every real-time PCR reaction was performed in duplicate (technical replicates). The specific melting curve of the amplified product carried out within protocol served as verification of the specifity in the PCR products. Results were collected using OneStep Software; Ct values were analysed comparing the average between control and ovariectomized samples, and obtaining the fold change values. *ACTB* and *GADPH* genes were used as control for the data normalization. Student’s *t*-test was calculated between both groups and in all cases the significance was statistically relevant (*p*-value < 0.05). All RT-PCR processes and reporting comply with MIQE guidelines [62].

## Abbreviations

ART, assisted reproductive technologies; DEGs, differentially expressed genes; GO, gene ontology GO; IPA, ingenuity pathway analysis; LIMMA, linear models for microarray data; miRNA, microRNA; RT-PCR, reverse transcription-PCR; UO, unilateral ovariectomy

## Additional files

Additional file 1: Table S1. Counter current ovarian exchange references. (DOCX 27 kb) Additional file 2: Table S2. Primer sequences used to amplify specific fragments of porcine transcripts. (DOCX 14 kb) Additional file 3: Table S3. Top molecular and cellular functions related to differentially expressed genes. (DOCX 14 kb)
