# Supplementary material for: The oviductal transcriptome is influenced by a local ovarian effect in the sow
Source: J Ovarian Res. 2016 Jul 22;9:44. doi: 10.1186/s13048-016-0252-9 (PMC4957888; doi:10.1186/s13048-016-0252-9)
Supplement: Additional file 1: Table S1. — Counter current ovarian exchange references. (DOCX 27 kb) [file 13048_2016_252_MOESM1_ESM.docx]

**S1 Table. Counter current ovarian exchange references**

| **SPECIES** | **OVIDUCT** | **UTERUS** |
| --- | --- | --- |
| Human | Oxytocin and vasopressin ([2](#_ENREF_2)) | Utero-ovarian blood vessels ([1](#_ENREF_1))  Oxytocin and vasopressin ([2](#_ENREF_2))  Myometrial contractions ([3](#_ENREF_3))  Sperm transport ([4](#_ENREF_4))  Steroid hormones ([5](#_ENREF_5)) |
| Pig | Prostaglandin ([6](#_ENREF_6), [7](#_ENREF_7))  Steroid hormones ([7](#_ENREF_7), [8](#_ENREF_8)) | Prostaglandin ([6](#_ENREF_6), [9](#_ENREF_9))  Steroid hormones ([8](#_ENREF_8), [10](#_ENREF_10))  Polypeptide hormones ([11](#_ENREF_11))  Cytokines ([12](#_ENREF_12)) |
| Mouse |  | Xenon ([13](#_ENREF_13)) |
| Hamster |  | Xenon ([13](#_ENREF_13)) |
| Sheep |  | Utero-ovarian blood vessels ([14](#_ENREF_14), [15](#_ENREF_15)) Prostaglandin ([16-18](#_ENREF_16)) |
| Cat |  | Progesterone ([19](#_ENREF_19)) |
| Rabbit | Heat ([20](#_ENREF_20)) |  |

1. Bendz A. Countercurrent exchange in the human female reproductive tract: a study on extrinsic utero-ovarian blood vessels1982.

2. Schaeffer JM, Liu J, Hsueh AJ, Yen SS. Presence of Oxytocin and Arginine Vasopressin in Human Ovary, Oviduct, and Follicular Fluid. The Journal of Clinical Endocrinology & Metabolism. 1984;59(5):970-3.

3. Lyons E, Taylor P, Zheng X, Ballard G, Levi C, Kredentser J. Characterization of subendometrial myometrial contractions throughout the menstrual cycle in normal fertile women. Fertility and sterility. 1991;55(4):771-4.

4. Kunz G, Herbertz M, Noe M, Leyendecker G. Sonographic evidence for the involvement of the utero-ovarian counter-current system in the ovarian control of directed uterine sperm transport. Human reproduction update. 1998;4(5):667-72.

5. Cicinelli E, Einer‐Jensen N, Barba B, Luisi D, Alfonso R, Tartagni M. Blood to the cornual area of the uterus is mainly supplied from the ovarian artery in the follicular phase and from the uterine artery in the luteal phase. Human reproduction. 2004;19(4):1003-8.

6. Stefanczyk-Krzymowska S, Chłopek J, Grzegorzewski W, Radomski M. Local transfer of prostaglandin E2 into the ovary and its retrograde transfer into the uterus in early pregnant sows. Experimental physiology. 2005;90(6):807-14.

7. Hunter RHF, Cook, NL P. Regulation of oviduct function in pigs by local transfer of ovarian steroids and prostaglandins: a mechanism to influence sperm transport. European Journal of Obstetrics & Gynecology and Reproductive Biology. 1983;14:225-32.

8. Stefańczyk-Krzymowska S, Grzegorzewski W, Wasowska B, Skipor J, Krzymowski T. Local increase of ovarian steroid hormone concentration in blood supplying the oviduct and uterus during early pregnancy of sows. Theriogenology. 1998;50(7):1071-80.

9. Gleeson A. Proceedings: Luteal function in the cyclic sow after infusion of prostaglandin F 2alpha through a uterine vein. Journal of reproduction and fertility. 1974;36(2):487.

10. Krzymowski T, Kotwica J, Stefańczyk S, Czarnocki J, Dȩbek J. A subovarian exchange mechanism for the countercurrent transfer of ovarian steroid hormones in the pig. Journal of reproduction and fertility. 1982;65(2):457-65.

11. Koziorowski M, Krzymowski T, Stefańczyk-Krzymowska S, Czarnocki J, Kotwica J, Ziecik A. Counter current transfer of polypeptide hormones from uterus to ovary in gilts using insulin as a model. Acta physiologica Polonica. 1988;39(2):121.

12. Waberski D, Dohring A, Ardón F, Ritter N, Zerbe H, Schuberth H-J, et al. Physiological routes from intra-uterine seminal contents to advancement of ovulation. Acta Vet Scand. 2006;48:13.

13. Einer-Jensen N. Local transfer of 133xenon from the uterine horn to the ipsilateral ovary in the mouse, hamster and guinea-pig. Journal of reproduction and fertility. 1974;40(2):479-82.

14. Staples L, Fleet I, Heap R. Anatomy of the utero-ovarian lymphatic network and the composition of afferent lymph in relation to the establishment of pregnancy in the sheep and goat. Journal of reproduction and fertility. 1982;64(2):409-20.

15. Zezula-Szpyra A, Gawrońska B, Skipor J. Vasa vasorum of blood and lymph vessels in the broad ligament of the sheep uterus analyzed by scanning electron microscopy. Roczniki Akademii Medycznej w Bialymstoku (1995). 1996;42:134-46.

16. McCracken J, Carlson J, Glew M, Goding J, Baird D, Gréen K, et al. Prostaglandin F 2 identified as a luteolytic hormone in sheep. Nat New Biol. 1972;238(83):129-34.

17. Mapletoft R, Ginther O. Adequacy of main uterine vein and the ovarian artery in the local venoarterial pathway for uterine-induced luteolysis in ewes. American journal of veterinary research. 1975;36(7):957-63.

18. Lamond D, Drost M. The counter-current transfer of prostaglandin in the ewe. Prostaglandins. 1973;3(5):691-5.

19. Bendz A, Lundgren O, Hamberger L. Countercurrent exchange of progesterone and antipyrine between human utero‐ovarian vessels, and of antipyrine between the femoral vessels in the cat. Acta Physiologica Scandinavica. 1982;114(4):611-6.

20. Bahat A, Eisenbach M, Tur-Kaspa I. Periovulatory increase in temperature difference within the rabbit oviduct. Human reproduction. 2005;20(8):2118-21.
