# Supplementary material for: The oviductal transcriptome is influenced by a local ovarian effect in the sow
Source: J Ovarian Res. 2016 Jul 22;9:44. doi: 10.1186/s13048-016-0252-9 (PMC4957888; doi:10.1186/s13048-016-0252-9)
Supplement: Additional file 2: Table S2. — Primer sequences used to amplify specific fragments of porcine transcripts. (DOCX 14 kb) [file 13048_2016_252_MOESM2_ESM.docx]

**S2 Table. Primer sequences used to amplify specific fragments of porcine transcripts.**

| **Gene symbol** | **PCR-product (bp)** | **Melting point (°C)** | **Primer sequence** | **Gene Bank accession** |
| --- | --- | --- | --- | --- |
| *ALOX12* | 97 | 84,5 | CACGACATGAAGCAAACCAC  AACCCTGACGCCAAATACCT | NM_213931 |
| *CFH* | 181 | 82,2 | CCTGCTCCAAGATGTACCTTGAAA  CATTCCGTTTGCACGTCAGG | NM_214281 |
| *SAL1* | 116 | 80,5 | AGCCTTTAAATTTCAGAGAAAGGT  GGCGAAATTTGTTCTCTCCA | NM_213814 |
| *OVGP1* | 143 | 83,8 | TCCCACATATGGACGGACTT  GGAGGTTGCTCACAGAAGGA | [NM_214070](http://www.ncbi.nlm.nih.gov/nuccore/NM_214070.1) |
