# Supplementary material for: The oviductal transcriptome is influenced by a local ovarian effect in the sow
Source: J Ovarian Res. 2016 Jul 22;9:44. doi: 10.1186/s13048-016-0252-9 (PMC4957888; doi:10.1186/s13048-016-0252-9)
Supplement: Additional file 3: Table S3. — Top molecular and cellular functions related to differentially expressed genes. (DOCX 14 kb) [file 13048_2016_252_MOESM3_ESM.docx]

**S3 Table. Top molecular and cellular functions related to differentially expressed genes.**

| **Molecular and cellular functions** | **Nº of molecules** | **Factors** |
| --- | --- | --- |
| Cell death and survival | 9 | *CHF, MTOR, SEPP1, ALOX12, SEMA3B, PRAP1, PTH1R, RBBP6, SEPP1* |
| Amino acid metabolism | 4 | *MTOR, SEPP1, ALOX12, CHF* |
| Small molecule biochemistry | 7 | *ALOX12, SEPP1, PTHR1, AKAP5, CHF, MTOR, ITIH2* |
| Carbohydrate metabolism | 4 | *MTOR, ITIH2, CHF, SEPP1* |
| Cell morphology | 7 | *PTH1R, ALOX12, MTOR, AKAP5, SEMA3B, CHF, SEPP1* |
| Cell-to-cell signalling | 5 | *IGHA1, MTOR, ALOX12, CHF, AKAP5* |
